# Differential transcriptional invasion signatures from patient derived organoid models define a functional prognostic tool for head and neck cancer

**DOI:** 10.1038/s41388-024-03091-4

**Published:** 2024-06-28

**Authors:** Peter D. Haughton, Wisse Haakma, Theofilos Chalkiadakis, Gerben E. Breimer, Else Driehuis, Hans Clevers, Stefan Willems, Stefan Prekovic, Patrick W. B. Derksen

**Affiliations:** 1 Department of Pathology, University Medical Center Utrecht, Utrecht, The Netherlands; 2 Center for Molecular Medicine, University Medical Center Utrecht, Utrecht, The Netherlands; 3 Hubrecht Institute, Royal Netherlands Academy of Arts and Sciences (KNAW) and UMC Utrecht, 3584 CT Utrecht, The Netherlands; 4 Roche Pharmaceutical Research and Early Development, Basel, Switzerland; 5 Department Pathology and Medical biology, University Medical Center Groningen, Groningen, The Netherlands

**Keywords:** Head and neck cancer, Transcriptomics, Extracellular matrix, Cancer models

## Abstract

Clinical outcome for patients suffering from HPV-negative head and neck squamous cell carcinoma (HNSCC) remains poor. This is mostly due to highly invasive tumors that cause loco-regional relapses after initial therapeutic intervention and metastatic outgrowth. The molecular pathways governing the detrimental invasive growth modes in HNSCC remain however understudied. Here, we have established HNSCC patient derived organoid (PDO) models that recapitulate 3-dimensional invasion in vitro. Single cell mRNA sequencing was applied to study the differences between non-invasive and invasive conditions, and in a collective versus single cell invading PDO model. Differential expression analysis under invasive conditions in Collagen gels reveals an overall upregulation of a YAP-centered transcriptional program, irrespective of the invasion mode. However, we find that collectively invading HNSCC PDO cells show elevated levels of YAP transcription targets when compared to single cell invasion. Also, collectively invading cells are characterized by increased nuclear translocation of YAP within the invasive strands, which coincides with Collagen-I matrix alignment at the invasive front. Using gene set enrichment analysis, we identify immune cell-like migratory pathways in the single cell invading HNSCC PDO, while collective invasion is characterized by overt upregulation of adhesion and migratory pathways. Lastly, based on clinical head and neck cancer cohorts, we demonstrate that the identified collective invasion signature provides a candidate prognostic platform for survival in HNSCC. By uncoupling collective and single cell invasive programs, we have established invasion signatures that may guide new therapeutic options.

## Introduction

HPV-negative Head and neck squamous cell carcinoma (HNSCC) constitutes almost 75% of all head and neck cancers of which 85% arise from the mucosal epithelial layers pertaining to the oral/nasal cavity, pharynx, and larynx [1, 2]. Five-year survival for locally advanced disease remains low at 35%, attributed to distant outgrowth recurrence and a propensity for tumor infiltration of the healthy surrounding tissue, mandible, and perineural structures [3]. Surgical intervention is often the first line of therapy for eligible patients, posing a risk of both functional and cosmetic disfigurement, with a notable 90% tumor recurrence rate attributed to inadequate surgical resection margins [4]. In particular, defining the pattern of invasive growth is a vital pathological parameter in HNSCC prognosis, as it can predict loco-regional relapse and lymph node metastasis. It is therefore imperative that the molecular programs controlling invasive growth are identified and established to aid future intervention and follow-up.

Distinct invasive morphological features in HNSCC have been defined as either well-delineated infiltrating borders, diffuse infiltrating solid cords or strands, or widespread cellular dissociation in small groups and/or single cells [5–7]. Consistently, pathological assessment of the deepest invasive cells using the invasive front grading (IFG) system dictates HNSCC patient outcome through exploiting morphological features such as keratinization, immune infiltration, nuclear polymorphism, and mode of invasion [5, 6]. Brandwein-Gensler et al*.* have evaluated patterns of invasion and developed the worst pattern of invasion (WPOI) consisting of broad tumor pushing margins that invade in solid cords and strands, invasive tumor islands of more than 15 cells, invasive tumor islands with less than 15 cells or tumor islands more than 1 mm positioned from the tumor invasion front. Importantly, WPOI assessment independently determines disease-free survival and overall survival, and can be used to predict outcome for patient with stage I-II HNSCC [8, 9].

Molecularly, it is well established that inter-cellular junctions dictate diverse phenotypic modes of invasion termed collective or single cell invasion, respectively. Recent efforts further defined single cell invasion as either mesenchymal, a mode specified by elevated cell-extracellular matrix (ECM) adhesion and proteolysis, or amoeboid, characterized by increase cortical actomyosin contractility [10–13]. However, most solid tumors invade collectively, navigating local tissues while maintaining adherens and tight junctions, which transduce mechanical and force transmission signals to regulate actomyosin dependent cytoskeletal rearrangement and cellular polarity [14, 15]. Importantly, identification of distinct cell populations exhibiting partial epithelial-to-mesenchymal transitions at the HNSCC invasive front, renders a binary EMT unlikely and indicate a void in invasive programs that control HNSCC invasion [16].

Tumor-stromal interactions predominantly govern local invasion across all cancer types through aberrant regulation of adhesion, degradation, and ECM remodeling molecules. Typically, cell-ECM adhesion receptors mainly through integrins, generate traction forces to migrate and invade surrounding tissue. YAP, a transcriptional co-activator, is a mechanosensitive transducer of extracellular cues that is actively translocated to the nucleus during collective invasion in invasive cancer models [17]. During this process, mechanical cues through integrin mediated ECM adhesion promote YAP activity, driving actomyosin contraction through transcription of Rho-GTPase activating proteins [18–20]. In HNSCC, nuclear YAP is predominantly found at the tumor invasive front and is correlated with aggressive-poorly differentiated HNSCC [21, 22]. The YAP transcriptional target cysteine-rich angiogenic inducer 61 (*CYR61*) has been shown to enhance HNSCC migratory pathways through ECM remodeling [23]. Conversely, connective tissue growth factor (CTGF), a YAP target that interacts with integrins, impairs HNSCC migration [24], indicating that YAP differentially regulates its targets during invasion.

To investigate which transcriptional programs dictate invasive growth patterns in head and neck cancer, we developed invasive HNSCC patient derived tumor organoid (PDO) models that recapitulate different 3D tissue invasion modes as observed in primary patient samples. We subjected collective and single cell invading models to single cell mRNA sequencing, which identifies elevated YAP activity as a main transcriptional target during collective invasion. We find that distinct collective and single cell invasive transcriptional signature show prognostic value in clinical HNSCC cohorts.

## Results

### HNSCC PDOs serve as a platform to study invasion

**Fig. 1:**
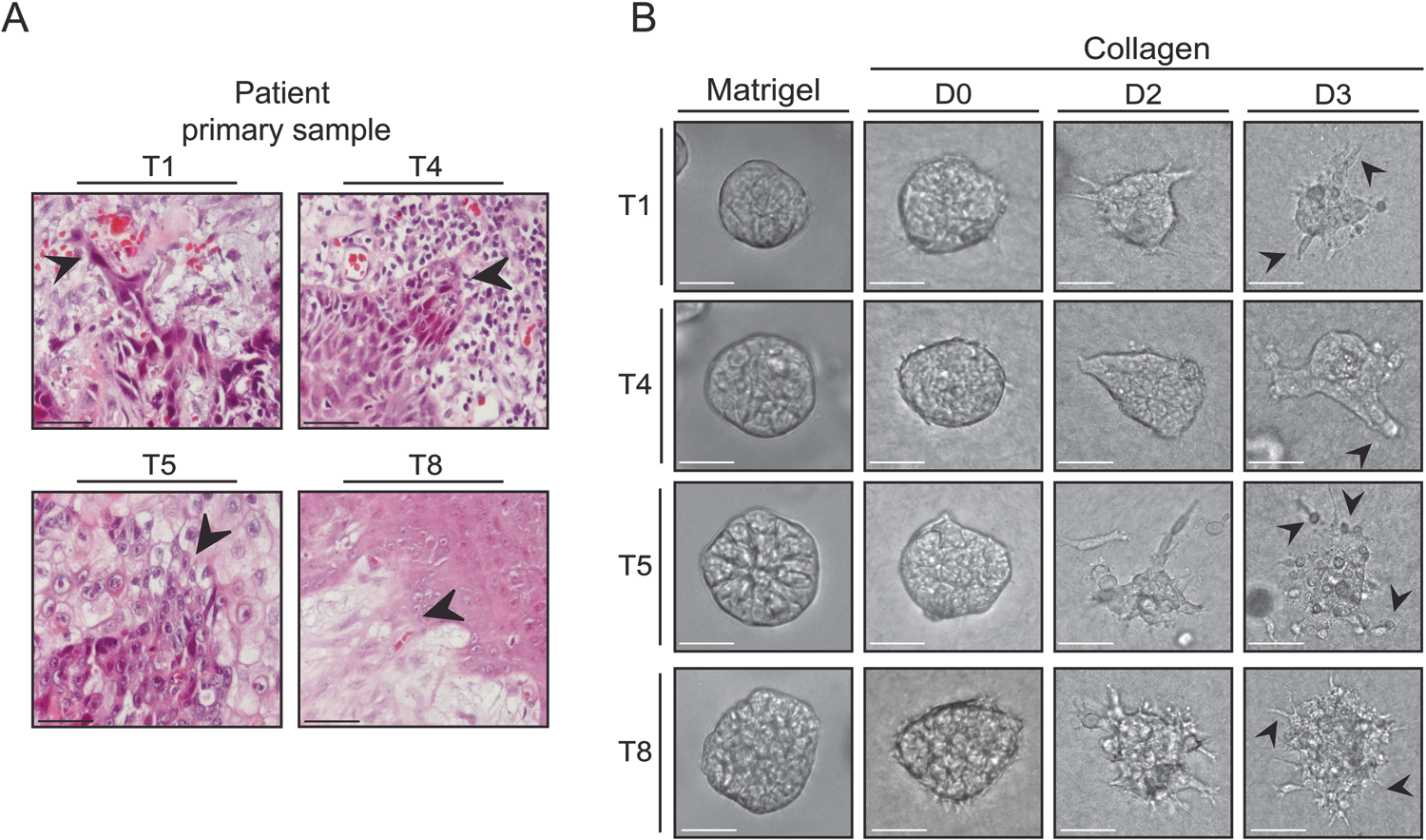
HNSCC PDOs serve as a platform to study modes of invasion. **A** Modes of invasion found in HNSCC. Panel of HNSCC primary tissue sections depicting single cell/cord like invasion (T1), collective invasion (T4), single cell invasion (T5), and collective/ protrusive invasion (T8). Black arrows indicate observable invasion. Scale bars: 20 µm. **B** HNSCC PDOs recapitulate invasive pattern found in corresponding primary sections. Brightfield images of PDOs cultured in Matrigel for 6 days or seeded in Collagen-I gels and imaged over 3 days. Black arrows indicate invasive structures. Scale bars: 100 µm.

Histomorphological assessment of 4 primary tissue sections from HNSCC patients, corroborates previous patterns of cancer cell invasion reported in the tumor invasive front grading system by Bryne et al. [5, 6]: Single cell/cord like (T1), collective multicellular strand (T4), single cell (T5) or collective strand/cord like/single cell (T8) (Fig. 1A). From these primary HNSCC samples we developed a panel of 8 PDO models that were propagated efficiently in Matrigel, a Laminin/Collagen-IV rich basement membrane extract, as previously described [25]. In Matrigel, the established PDO models do not exhibit invasion (Fig. 1B; left panels). However, when seeded in a pro-invasive Collagen type 1 matrix (Collagen pre-polymerized at 26 °C), we observed invasive patterns that are similar to the primary tissue sections (Fig. 1A, B), including expression of Keratin 14 (K14) a basal maker for poorly differentiated tumors and the tumor invasive front HNSCC. Interestingly, K14 expression did not show an expression bias towards invasive modes in the PDOs or their corresponding primary tissues (Supplementary Fig. 1A), indicating that a more complex cellular wiring creates diverse invasion. We also assessed the invasive patterns of four additional HNSCC PDOs (T2, T6, T258 and T276) but did not detect explicit instances of collective or single cell invasion (Supplementary Fig. 1B).

In order to further investigate exclusive invasion modes, we omitted HUB-T1 and HUB-T8 as they exhibit a combination of single cell and collective invasive patterns, leaving HUB-T4 (T4) and HUB-T5 (T5) for further analyses. PDO T4 shows uniform collective invasion, whereas PDO T5 shows exclusively single cell invasion (Fig. 1B).

### Collagen drives a YAP associated transcriptional program in HNSCC during invasion

**Fig. 2:**
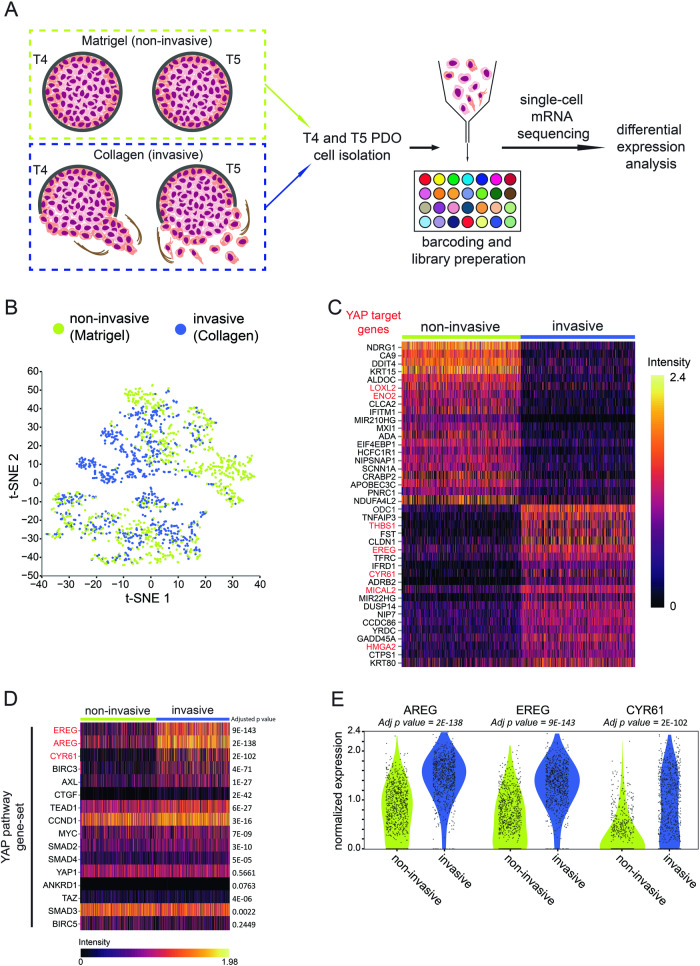
Single cell mRNA sequencing reveals unique YAP and downstream target transcription profiles of HNSCC PDOs seeded in Collagen matrices. **A** Schematic showing single cell mRNA sequencing of T4 and T5 HNSCC PDOs seeded in either Matrigel (termed ‘non-invasive’) or Collagen (termed ‘invasive’) and subsequent downstream analysis. **B** Single cell mRNA sequencing of collective and single cell models seeded in non-invasive or invasive matrix. A t-distributed stochastic neighbor embedding (t-SNE) plot was used to visualize the data. **C** Unique transcription profiles in invasive and non-invasive conditions. Top 20 differentially expressed (DE) markers associated with invasive and non-invasive conditions. Scaled expression displayed using a heatmap. Highlighted in red are established YAP-TEAD target genes. **D** Invasion into Collagen-I matrices in 3D leads to upregulation of YAP associated genes. Heatmap displays scaled expression of YAP/TAZ and their downstream targets of PDOs seeded in invasive and non-invasive conditions. Adjusted *p* value is displayed on the right. Highlighted in red are genes with a logFC ≥ 0.5. **E** Violin plots of significantly DE YAP downstream targets identified in invasive *versus* non-invasive HNSCC PDO models.

Recent single cell transcriptomic advancements have demonstrated tumor ecosystem complexity through profiling of the microenvironment and malignant cell populations [16]. However, due to an abundance of extrinsic factors, the exact molecular programs induced by ECM-independent interactions to control invasion remain unclear. To establish a general invasive transcriptional program irrespective of invasive mode, we first assessed differentially expressed (DE) genes in the T4 and T5 models when seeded in non-invasive (Matrigel) versus invasive (Collagen type-I) conditions, and performed single cell mRNA sequencing (Fig. 2A). Cells with low read counts and high mitochondrial expression were removed from further analysis (Supplementary Fig. 2A–C). Next, data were integrated and dimensionality reduction was performed on pooled T4 Matrigel (*n* = 545) and T5 Matrigel (*n* = 379) samples, now collectively termed ‘non-invasive’, and T4 Collagen (*n* = 509) and T5 Collagen (*n* = 386), henceforth designated ‘invasive’ (Fig. 2B). We confirmed that both PDO models are derived from an epithelial origin using previously established markers [16] (Supplementary Fig. 2D).

Although unbiased clustering using a Louvain algorithm resolved 12 clusters, pinpointing the invasive populations was challenging and would have required clear distinction from non-invasive cell-ECM cells and core cells (not in contact with the ECM) (Supplementary Fig. 2E). Because we did not identify cell populations that showed distinct expression of recognized markers for invasion such as *KRT14* [26], *DDR1* [27], or *ITGA2* [28, 29], further downstream analysis was performed on a sample comparison basis (Supplementary Fig. 2F–H). Subsequent DE analysis revealed 65 genes upregulated in non-invasive (logFC ≥ 0.5*,* adj *p* < 0.001), and 50 genes upregulated in invasive (logFC ≥ 0.5*,* adj *p* < 0.001) conditions (Supplementary Table 1). Closer inspection of the invasive top 20 differentially expressed genes showed an enrichment in pro-invasive YAP-associated genes such as *THBS1*, *EREG*, *CYR61*, *MICAL2,* and *HMGA2* (Fig. 2C, genes highlighted in red). In contrast, the YAP target genes *LOXL2* and *ENO2* were differentially upregulated in non-invasive samples (Fig. 2C, YAP target genes highlighted in red). Our findings that YAP targets are actively transcribed (Fig. 2D, E), suggest that pro-invasive Collagen induces nuclear translocation of YAP in HNSCC.

### Distinct molecular signatures define invasion modes of HNSCC

**Fig. 3:**
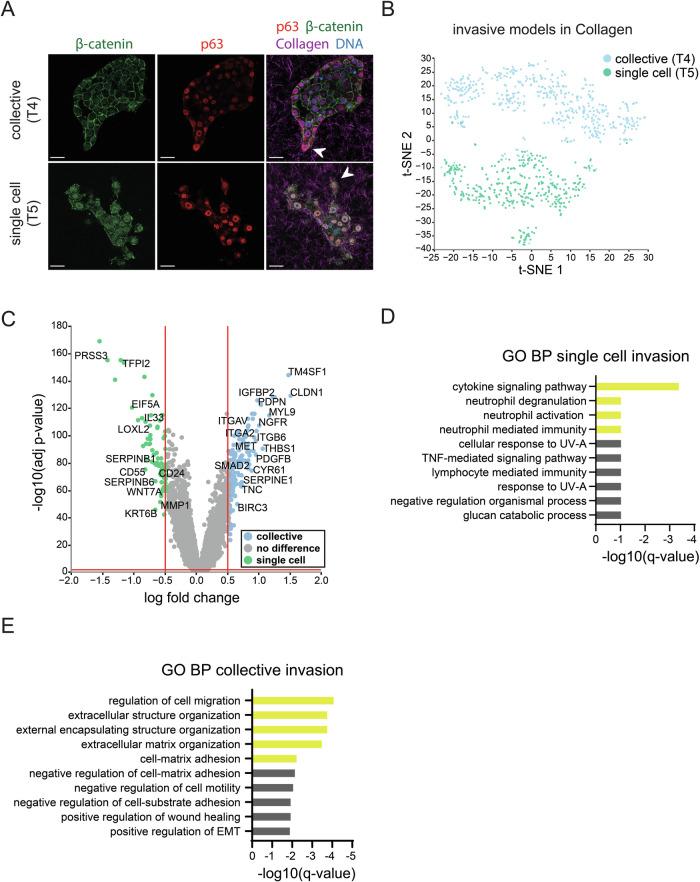
Distinct transcriptomic profiles define collective and single cell HNSCC PDO invasive models. **A** Selected T4 (henceforth termed the ‘collective’ model) and T5 (henceforth termed the ‘single cell’ model) Collagen-embedded PDOs were immunofluorescence labeled with β-catenin (green), p63 (Basal marker; red). Merged image with DNA (blue) and Collagen-I (magenta) are shown in the right panel. Arrows indicate invasive strands or single cells in the respective models. Scale bars: 50 µm. **B** Data integration and dimensionality reduction of transcriptomic data from single cell and collective invasive models seeded in Collagen. A t-SNE plot was used to visualize the data. **C** Volcano plot of DE genes comparing single cell vs collective models. Vertical red lines indicate logFC ≥ 0.5 and logFC ≤ −0.5 cut-offs. Horizontal red line indicates adjusted *p* value cut-off (<0.001). Both aforementioned logFC and adjusted *p* value were designated as differentially expressed (DE) genes. Labeled are selected genes linked to invasion from literature. **D** GSEA identifies unique molecular pathways in single cell invasion model. Bar graph depicts biological processes enriched gene ontology (GO) terms in the single cell invasion model. X-axis displays genes and y-axis top 10 enriched pathways. Highlighted in yellow are pathways of interest. **E** GSEA identifies unique molecular pathways in the collective invasion model. Bar graph depicts biological processes enriched GO term in the collective invasion model. Highlighted in yellow are pathways significantly enriched.

Morphological stratification of invasive patterns that are used to predict patient outcome in HNSCC suggests that there may be clinical benefit in identifying underlying molecular invasive programs [5, 30]. Therefore, to further characterize invasive T4 and T5 models, henceforth defined as the ‘collective’ and ‘single cell’ invasive PDO models respectively, immunofluorescence was performed confirming basal characteristics of both models through expression of the marker p63 (Fig. 3A). Moreover, the collective model displayed coherent plasma membrane localized β-catenin expression, indicative of mature epithelial cell–cell junctions (Fig. 3A, upper panels). Conversely, the single cell model showed punctate β-catenin plasma membrane expression and nuclear β-catenin localization, suggesting loss of cell to cell contacts and activation of canonical Wnt signals (Fig. 3A, lower panels) [31]. These findings in the respective models were validated in the corresponding patient tissue (Supplementary Fig. 3A).

To identify transcriptional differences between collective and single cell invasion, the ‘invasive’ mRNA sequencing data were extracted and dimensions reduced to visualize the underlying local structure (Fig. 3B). Although 9 clusters were identified, no clear cluster pertaining to the collective strand or invasive single cells was apparent (Supplementary Fig. 3B). Therefore, subsequent comparative analysis was performed on all cells corresponding to the collective and single cell invasive models. Based on DE analysis, we observed that 76 genes are significantly upregulated during single cell invasion (logFC ≥ 0.5*,* adj *p* < 0.001) versus 111 genes during collective invasion (logFC ≥ 0.5*,* adj *p* < 0.001) (Fig. 3C and Supplementary Table 2). Next, we employed GSEA analyses on the DE genes to uncover biological pathways associated with each invasion pattern. Interestingly, cytokine-mediated signaling (adj *p* value 3.85E-04) was enriched in single cell invasion and, albeit non-significant, we noted an association with neutrophil activity *(*adj *p* value 8.66E-02) (Fig. 3D and Supplementary Table 3). Intuitively, this suggests that HNSCC single cell invasion may resemble pathways closer to immune migration. Indeed, several genes associated with neutrophile regulation found in the transcriptomic analysis associate with invasion such as *PYGB* (logFC = 0.6837, adj *p* value 1.64E-79), *SERPINB1* (logFC = 0.7123, adj *p* value 2.90E-62), *PRSS3* (logFC = 1.461, adj *p* value 8.50E-156), and *SERPINB6* (logFC = 0.5188, adj *p* value 1.06E-80) (Supplementary Tables 2 and 3).

During collective invasion, pathways contributing to regulation of cell migration (adj *p* value 7.60E-05), extracellular matrix organization (adj *p* value 1.58E-04) and cell-matrix adhesion (adj *p* value 0.005346) were significantly upregulated (Fig. 3E and Supplementary Table 4). Therefore, we also analyzed integrin expression, which revealed an increase in *ITGA2* (logFC = 0.7003, adj *p* value 7.28E-98)*, ITGAV* (logFC = 0.7861, adj *p* value 3.48E-107) and *ITGB6* (logFC = 0.9132, adj *p* value 8.22E-97) in the collective model (Supplementary Fig. 3C). We found that *ITGB4* (logFC = 0.3543, adj *p* value 4.56E-68) was significantly higher expressed in the single cell invading model T5 (Supplementary Fig. 3B). Although several ECM remodeling and constituents were differentially regulated between both models, we noted higher expression of *FBLN1*, *LAMB1, TGFB1* and *PDPN* in T4, while T5 expressed higher levels of *APBB2, ENG, LOXL2, SULF2* and *WDR72* (Supplementary Fig. 3D, E). Together, these data suggest that collective and single cell invasion maintain unique invasive programs, and that collective invasive programs adhere closer to publicly consolidated GO pathways.

### Collective invasion correlates with increased YAP activity in HNSCC

**Fig. 4:**
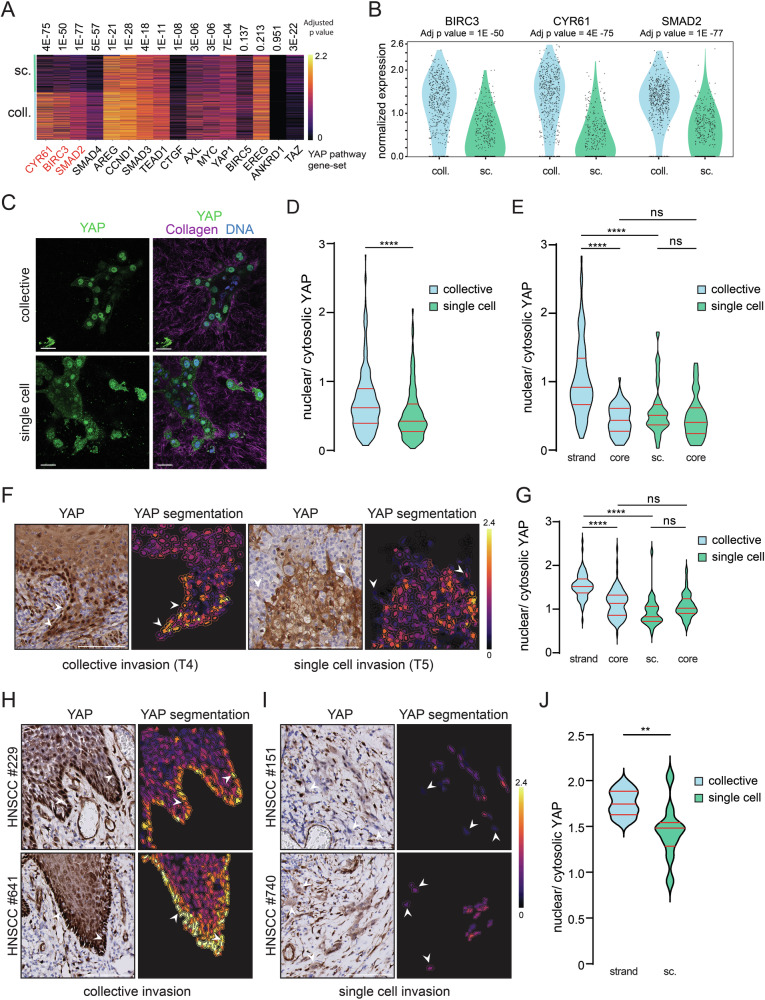
Collective invasion shows activation of YAP in the invasive strand in HNSCC. **A** Collective invasion in 3D leads to upregulation of YAP associated genes. Heatmap shows YAP/TAZ and downstream targets expression in collective (coll.) and single cell (sc.) invasion models. Adjusted *p* values are displayed above the heatmap. Highlighted in red are genes with a logFC ≥ 0.5. Signal intensity is indicated on the right. **B** Violin plots showing the significant DE YAP associated genes identified in collective versus single cell invasion. **C** Collective and single cell invasion models seeded in Collagen-I were Immunofluorescence labeled for YAP (green, left panels). Merged image with Collagen (purple) and DAPI (blue) and are shown in the right panels. Scale bars: 50 µm. **D** Quantification of nuclear-cytosolic ratios of YAP from 12 individual organoid structures in the collective and single cell invasive models shown in (**C**). **E** Nuclear-cytosolic ratios of YAP in collective strands or invasive single cells versus PDO cores (inner cell population) from both models. Blue represents the collective models and green the single cell invasive model. Twelve individual organoid structures were quantified. **F**, **G** YAP expression was assessed using IHC and the subcellular localization was analyzed and visualized using a pseudo color overlay of cellular mean YAP-DAB intensity at the tumor invasive front (YAP segmentation). Arrows indicate invasive structures. Signal intensity is indicated on the right. Scale Bars: 100 µm **F** The nuclear/cytosolic YAP ratio was quantified in five individual regions (62,500 µm^2^ each) per sample in the collectively invasive strands, single cells, or tumor core, in the respective models (**G**). **H**–**J** YAP activity is increased in collective HNSCC invasion. Representative examples from an independent cohort of collective (**H**) and single cell (**I**) invasive HNSCC samples were IHC stained for YAP protein expression and analyzed as in (**F**, **G**). Nuclear/cytosolic YAP ratios were quantified as in (**F**, **G**) for 10 collectively invading HNSCC samples and 10 invasive single cell HNSCC samples (**J**). Error bars (red dashed lines) indicate standard deviation; ns non-significant; **p < 0.01, ****p < 0.001. Statistical significance was calculated using the unpaired *t* test (**D**, **J**) or Kruskal–Wallis test (**E**, **G**).

Because our data indicate that invasion promotes activation of transcriptional YAP targets and collective invasion strongly associates with cell-ECM adhesion pathways, we investigated if collective and single cell invasion modes induce differential regulation of established YAP transcriptional targets. Whereas most examined YAP targets were expressed similarly between both models, we observed that *BIRC3, CYR61*, and *SMAD2* showed a significant increase in expression, specifically in the collective invasive model (Fig. 4A, B). To study how collective and single cell invasion coincide with spatial YAP protein expression, we probed protein localization of YAP during invasion in 3D Collagen. In agreement with the mRNA expression profiles, we observe that both models express nuclear YAP (Fig. 4C). Indeed, YAP nuclear translocation is elevated in the collective invasion model compared to the single cell invasion model (Fig. 4D). Additionally, YAP activation is increased in invasive strands compared to both the core of the collective model and invasive single cells (Fig. 4E), an observation that was also found in the corresponding primary tissue of the PDO models (Fig. 4F, G). To further substantiate the clinical ramifications of these findings, whole tissue sections from an independent cohort of collective or single cell invasive HNSCC samples were analyzed for YAP localization, which confirmed elevated YAP activity in the collectively invading strands but not in the single cell invading tumors (Fig. 4H–J). Because elevated nuclear YAP localization in the invasive strands also coincides with increased aligned Collagen fibers up to 100 µm (Supplementary Fig. 4A, B), it suggests that collectively invading cells induce increased cell-to-ECM mechanical forces to enable regional invasion.

In short, our data indicate that YAP activity is mainly dictated by concomitant cell–cell and cell-ECM mechano-transduction cues, which subsequently promote collagen alignment during collective invasion.

### The Collective invasion signature associates with decreased survival in cancer

**Fig. 5:**
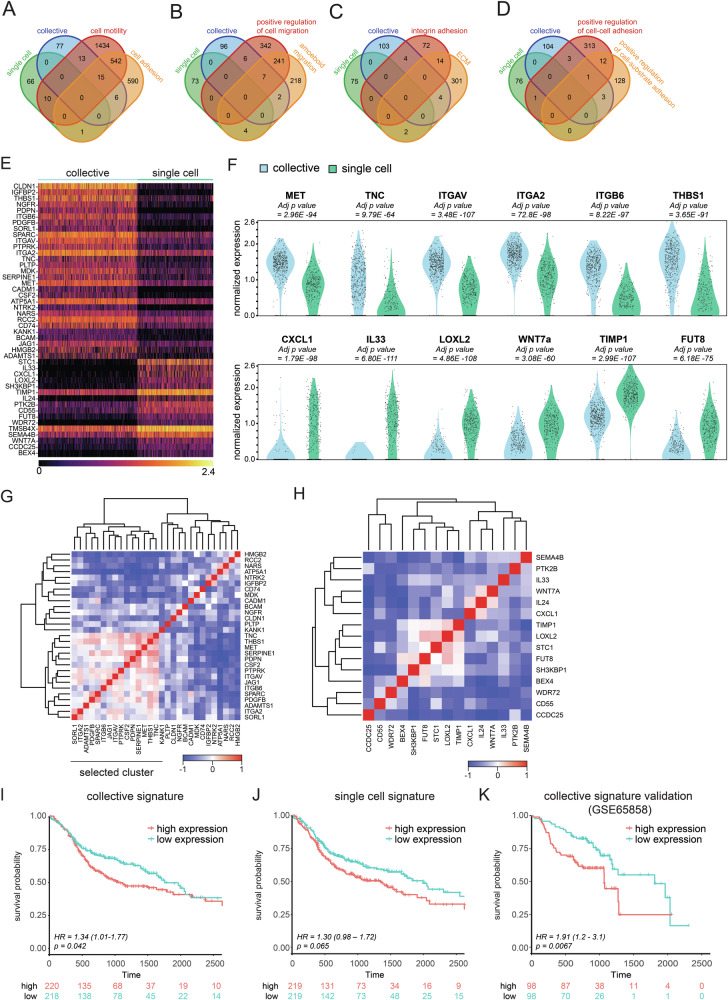
High expression of the collective invasion signature associates with decreased patient survival in HNSCC. **A** Publicly curated adhesion and motility gene-sets associate with model specific invasion. Venn diagram showing a total of 2014 GO cell motility and 1154 GO cell adhesion data sets, cross-compared with the number of DE genes derived from single cell versus collective invasive models. Venn diagrams showing enriched collective and single cell invasion genes that overlap with subdivision terms of ‘cell motility’ and ‘cell adhesion’: “positive regulation of cell migration’ and ‘amoeboid-like migration’ (**B**), ‘integrin adhesion’ and ‘ECM’ (**C**), ‘positive regulation of cell–cell adhesion’ and positive regulation of cell-substrate adhesion” (**D**). **E** Transcriptional signatures associated with collective and single cell invasion in HNSCC PDOs. Heatmap shows expression of overlapping DE genes associated with motility and adhesion GO terms for collective and single cell invasion. **F** Selected DE genes from either the collective (upper panel) or single cell (lower panel) signature. Violin graphs depict normalized expression on the y-axis and samples on the x-axis. **G**, **H** Single cell signature gene do not show clear cross-correlation with the head and neck cancer TCGA database whereas the collective signature reveals distinct cluster. Heatmaps depict hierarchical clustering to identify genes with strong cross-correlation patterns. **G** The collective gene signature was specified by selecting highly cross correlative genes. Clustered genes were selected for further analysis. **H** All single cell signature genes were used for further analysis as no clear cross correlation was determined. **I**, **J** High expression of the collective signature has prognostic value in HNSCC. Kaplan–Meier survival curves of collective (**I**, **K**) and single cell signature data (**J**) from head and neck cancer TCGA cohort (**I**, **J**, *n* = 438) and GSE65858 (K, *n* = 196). The two groups, defined by z-scores, were compared using a log-rank test. Survival curves depicts the probability of survival over time, where the x-axis represents time in days, and the y-axis indicates the proportion of patients who survived. Statistical significance was determined by *p* value calculation, showing any potential differences in OS between the two groups. Hazard ratios (HRs) and 95% confidence intervals for survival curves were calculated using a Cox proportional hazards model. Risk table was included, providing the number of patients at risk at different time points.

To expand on migratory and adhesion GSEA pathways and probe potential clinical relevance, we extracted all logFC *≥* 0.5 genes (adj *p* < 0.001) in both models, and cross-compared matching genes with all publicly available GO cell motility (2014 genes) and cell-adhesion (1154 genes) datasets (Fig. 5A). As expected, we observed an increase in the transcription of genes associated with motility and adhesion in the collectively invading model compared to single cell invasion (Fig. 5A). Further examination of sub-GO terms of motility and cell adhesion suggest that the single cell model contains characteristics of amoeboid-like migration (Fig. 5B), whereas the collective model featured processes like positive regulation of cell migration, cell–cell adhesion, and cell-substrate adhesion (Fig. 5C, D). Based on these data, we selected the genes specific to either mode of invasion that were upregulated in migratory and adhesion GSEA pathways, and generated a collective and single cell invasive gene signature (Fig. 5E, F).

We next analyzed potential clinical prognostic power of the HNSCC PDO collective and single cell invasive gene signatures by examining gene cross-correlations in the TCGA head and neck cancer cohort, which resulted in a final selection of 15 clustered genes within the collective invasion signature (Fig. 5G). Because no clear correlated cluster was identified for the single cell invasion signature and no match with TMS4BX was found in the head and neck cohort, all remaining genes were defined as the single cell invasion signature (Fig. 5H). As such, we define a new unique collective and single cell invasive signature, designated “COL_H&NINV” and “SC_H&NINV” (Table 1). Next, we investigated if the defined gene signatures associate with survival in the TCGA head and neck cancer cohort. Interestingly, high expression of the collective gene invasion signature associates with poor survival (Hazard ratio (HR): 1.34, 95% confidence interval (CI): 1.01–1.77, *p* = 0.042) (Fig. 5I), while the single cell signature does not (HR: 1.3, CI: 0.98–1.72, *p* = 0.065) (Fig. 5J). Importantly, we validated the collective signature in an independent HNSCC cohort (GSE65858 [32]), analyzing the HPV negative samples (*n* = 196)(HR: 1.91, 95% CI: 1.2–3.1, *p* = 0.0067) (Fig. 5K). To determine the general applicability of the collective signature as a prognosticator in other cancer groups, we analyzed all solid cancer types in the TCGA database. High expression of the collective signature indicated a declining survival trend in 18 out of 29 TCGA solid cancer groups (excluding head and neck cancer) (Supplementary Fig. 4A). Furthermore, 7 cancer types are prognostically significant using the collective invasive signature, of which 6 cancer types reached 50% survival probability in at least one condition (Supplementary Fig. 5B–H). Our results therefore show that the collective invasion signature shows promise as a prognostic biomarker in Head and Neck cancer that can potentially be translated to other invasive cancer types.

**Table 1:** Collective and single cell invasive signature in H&N cancer.

COL_H&NINV_Signature	SC_H&NINV_Signature
SORL1	STC1
ITGA2	IL33
ADAMTS1	CXCL1
PDGFB	LOXL2
SPARC	SH3KBP1
ITGB6	TIMP1
JAG1	IL24
ITGAV	PTK2B
PTPRK	CD55
CSF2	FUT8
PDPN	WDR72
SERPINE1	SEMA4B
MET	WNT7A
THBS1	CCDC25
TNC	BEX4

In sum, we have developed PDOs from patients with HNSCC that show differential invasion patterns in 3D Collagen matrices. Collective invasion in HNSCC points to a YAP-centered invasion signature that shows prognostic value in head and neck cancer.

## Discussion

Although invasive morphological patterns have been used as a prognosticator in HNSCC [8, 9], the differential expression regulating mode of invasion has remained unclear. Furthermore, good organotypic models of HNSCC that show invasion in 3D matrices were lacking until now. Here, we developed and employed invasive PDO models of HNSCC to investigate the molecular programs controlling collective or single cell invasion phenotypes. Our HNSCC PDO models represent the various modes of HNSCC invasion reported previously [5, 6]. Importantly, the pattern of invasion can predict patient survival and can be used to determine patient prognosis in low-stage OSCC [9, 33]. Our study agrees with these initial findings.

To be able to discriminate between invasion modes, we have opted to compare two PDO model systems, which displayed a pure and homogeneous invasion pattern; i.e., either collective or single cell invasion in 3D. Despite this reductionist approach, our key findings indicate that our approach yields a clear differential prognostic readout for the collective invasive signature. Our data show that collective invasion exhibits a more ‘conventional’ invasive program through upregulation of pre-established cell motility and adhesion genes, as opposed to single cell invasion. The transcriptional upregulation of *ITGB6*, *BCAM,* and *TGFBI*, in combination with coherent plasma membrane β-catenin localization, p63 expression, and nuclear translocation of YAP, suggests a basal program controls collective invasion in HNSCC. Paradoxically, an enrichment in multiple YAP associated genes and identification of “positive regulation of epithelial to mesenchymal transition” GSEA GO term in the collective model indicate an active EMT program. Interestingly, *MET* was upregulated in the collective invasive model, which has previously been shown to induce mesenchymal-type collective cell invasion when stimulated with its cognate ligand hepatocyte growth factor while maintaining cell adherence junctions [34]. In line with this, CLDN1 overexpression associates with high grade OSCC perineural invasion and shown to upregulate EMT marker Vimentin in conjunction with nuclear translocation of β-catenin in nasopharyngeal carcinoma [35, 36]. Conversely, overexpression of CLDN1 in triple negative “claudin 1 low” in the basal-like breast cancer cell line MDA-MB-231 inhibits cell migration and results in elevated cell adhesion with β-catenin localization to adherence junctions [37]. Interestingly, the collective HNSCC PDO model T4 would appear to exhibit traits of both profiles; coherent plasma membrane β-catenin and CLDN1 expression during invasion. Together, our data therefore indicate that a partial EMT-like state may be controlling HNSCC collective invasion [16]. Indeed, our collective signature reveals a combination of established EMT genes such as *PDPN*, *TGFBI,* and *TNC*, which have all been linked to poor prognosis in HNSCC patients [38, 39]. Interestingly, tumor budding, a morphological phenotype of multicellular dissociation, also exhibits a partial EMT profile, suggesting that the collective invasive signature may coincide with the tumor budding phenomenon [40, 41].

The YAP dependent Hippo pathway is controlled through extracellular stimulation, mechanical cues, cell–cell adhesion, and cell-ECM interactions [42], suggesting that cell–cell adhesion sequestering of YAP is bypassed by pro-nuclear YAP translocation signals mediated by cell-ECM interactions. Closer inspection of the collective model indeed shows increased Collagen-I alignment at the invasive front, suggesting that activation of Collagen and/or RGD receptor mediated mechanical force transduction and/or cell-ECM interactions are dominant regulators of YAP nuclear translocation in collective invasion. Our transcriptional analyses align with this, showing high expression of RGD and Collagen receptors *ITGAV*, *ITGB6,* and *ITGA2* [43–45] and secreted glycoproteins that enhance integrin-ECM interaction such as Thrombospondin-1 (*THBS1*) and Tenascin C (*TNC*). Interestingly, THBS1 promotes nuclear shuttling of YAP through direct interaction with integrin αvβ1, and has been identified as a YAP transcriptional target [46, 47]. Conversely, TNC can promote invasion through various integrins mediating YAP Inhibition [48, 49], while interactions with integrin αvβ6 or αvβ1 induce EMT like programs and nuclear translocation of β-catenin [50, 51]. This suggests that differential regulation of secreted glycoproteins and integrins direct actin cytoskeleton dynamics through focal adhesion complexes to generate a YAP positive feedback loop during collective invasion. The absence of distinct migratory or invasion signatures in single cell invasion using GSEA analysis indicates single cell invasion in HNSCC is poorly defined, and/or that single cell invasion may not express signatures that overlap with the published cell invasion programs. However, we found that several immune and cytokine related GO terminologies in the single cell invasive model overlapped with cell migration (GO:0016477). Chemokine (C-X-C motif) ligand 1 (CXCL1), a chemoattractant for neutrophils, can be secreted by cancer associated fibroblasts (CAFs), promoting ECM degradation and modulation of MMP secretion during cancer invasion [52]. More recently, independent of stomal immune cell influences, CXCL1 can stimulate invasion through ERK-MMP2/9 mediated ECM degradation and regulate cytoskeletal dynamics via Rho GTPase activity [53, 54]. Similarly, Interleukin 33 (IL-33) is secreted by CAFs during tumor progression but has also been found to enhance HNSCC invasion through MMP2/9 mediated ECM degradation [55]. Interestingly, single cell transcriptomics performed on HNSCC CAFs identified key cluster markers associated with MMP1, IL24 and CXCL1 [56]. Together, these data suggest that HNSCC single cell invasion may resemble an immune or CAF-like migratory program.

Here, we established a panel of invasive HNSCC PDO models that show a YAP-centered invasive program in 3D Collagen matrices, and identified a collective transcriptional signature that shows associations with poor prognosis in head and neck cancer. Our study represents an important step forward in understanding the molecular mechanism governing carcinoma invasion and linking specific invasion modes to cancer prognosis.

## Materials and methods

### Antibodies and reagents

The following primary antibodies were used: rabbit anti-human YAP (1:200, CST, catalog no. 4912), rabbit anti-human K14 (1:200, BioLegend, catalog no. 905301), mouse anti-p63 (1:50, Agilent Technologies, catalog no. m7317) and β-catenin (1:100, BD Biosciences, catalog no. 610154). Collagen was visualized by using either DIC internal reflection within the confocal microscope or GFP-CNA35 probe (1:1000) [17]. The following secondary antibodies were used for immunofluorescence: Alexa-fluor-488/568/647-conjugated goat anti-mouse or goat anti-rabbit antibodies (1:500, Invitrogen). To visualize the nucleus 4′,6-diamidino-2-phenylindole (DAPI, Thermo Fisher) was used. Brightvision goat anti-rabbit HRP (Cat# DPVR-HRP, Immunologic) and BrightDAB (Cat# BS04, Immunologic) was used for immunohistochemical assessment.

### PDO culture

HPV-negative HNSCC PDOs T1 (tongue squamous cell carcinoma (TSCC)), T2 (larynx SCC), T4 (TSCC), T5 (parotid gland SCC), T6 (oral cavity SCC), T8 (gingiva SCC), 258 T (TSCC) and 276 T (larynx SCC) were supplied and cultured according to pre-established protocols [25]. Briefly, PDOs were cultured and maintained in Matrigel (Basement membrane matrix, Corning, Cat# 356231) and incubated at 37 °C in 5% CO2. PDOs were passaged every 10 days using a combination of trypsin-EDTA (TrypLE Express, Cat# 12605-010) for 10 min. and mechanical sheering using a 1 mL pipette followed by an ice-cold PBS wash and centrifugation (1500 RPM, 5 min). Expansion media consisting of advanced DMEM F-12 (Thermo Fisher Scientific) medium containing 1% L-glutamine, 1% PenStrep and 1% HEPES buffer (Thermo Fisher Scientific) that was supplemented with 1X B27 (Invitrogen Life Technologies, catalog no. 17504-044), 1.25 mmol/L N-acetyl-L-cysteine (Sigma-Aldrich, catalog no. A9165), 10 mmol/L Nicotinamide (Sigma-Aldrich, catalog no. N0636), 0.3 μmol/L CHIR 99021 (Sigma-Aldrich, catalog no. SML1046), 1 μmol/L Prostaglandin E2 (Tocris Bioscience, catalog no. 2296), 50 ng/mL human EGF (PeproTech, catalog no. AF-100-15), 500 nmol/L A83-01 (Sigma, catalog no. SML0788-5MG), 5 ng/mL human FGF2 (PeproTech, catalog no. 100-18B), 1 μmol/L Forskolin (R&D Systems, catalog no. 1099), 10 ng/mL human FGF10 (PeproTech, catalog no. 100-26), 4% R-spondin and 4% Noggin (both produced via the r-PEX protein expression platform at U-Protein Express BV).

### Invasion assays

PDOs were cultured for 6 days in Matrigel, treated with 1 mg/mL of Dispase II (catalog no. 17105041, Life Technologies) to isolate single cells and washed three times in Ice cold PBS. To maximize invasion, Collagen networks (2 mg/mL final concentration) were pre-polymerized at 26 °C as previously reported [17]. Briefly, Collagen matrices were prepared using rat-tail Collagen (catalog no. 354236, Corning, Thermo Fisher), 10X phosphate buffer saline (PBS) (Gibco), NaOH and dH_2_O according to the manufacturer’s instructions. Next, PDOs were added to the Collagen mix (1/4 of total volume) and seeded in a pre-warmed (48 h at 26 °C) culture plate. Culture plates were inverted several times for 8 min. until homogeneous PDO distribution and network polymerization. Hereafter, the plates were incubated for 75 min. at 26 °C, media were added, and PDO models were allowed to invade for 72 h. Optimal invasion media consisted of DMEM F-12 (Thermo Fisher Scientific) medium containing 1% L-glutamine, 1% PenStrep, 1% HEPES buffer (Thermo Fisher Scientific) and 200 ng/mL human EGF (PeproTech, catalog no. AF-100-15). PDOs were imaged using phase contrast on the EVOS M5000 (Thermo Fisher Scientific) microscope or processed further for immunofluorescence imaging using confocal microscopy or single cell mRNA sequencing.

### Immunofluorescence and Immunohistochemistry (IHC)

PDOs embedded in Collagen type 1 were fixed with 4% paraformaldehyde (PFA; Sigma-Aldrich) for 15 min at room temperature and washed 3 times in 1x PBS. Fixed samples were blocked with 10% normal goat serum (Gibco) in 0.3% Triton-X (Sigma-Aldrich) for 1 h. Primary antibodies were diluted in antibody buffer (0.3% Triton-X with 1% w/v BSA in 1x PBS) and gels were incubated overnight at 4 °C with gentle agitation. Primary antibody was washed 4 times with 1x PBS (10–15 min each) and conjugated secondary antibodies combined with DAPI (5 µg/mL) were incubated overnight at 4 °C while shaking. Probed gels were washed and imaged on a Zeiss LSM 880 confocal microscope (40X objective, NA = 1.1). For immunohistochemistry, 4 μm tissue sections were deparaffinized and rehydrated with xylene, ethanol, and deionized H_2_O. Next, peroxidases were blocked for 15 min. with 1.5% hydrogen peroxide. Antigen retrieval was carried out by boiling for 20 min. in a pH 9.0 Tris-EDTA buffer, followed by cooling the sections and blocking with 1% BSA for 30 min. Primary antibody incubation was performed for 1 hr. at room temperature; sections were then washed with PBS and secondary antibody incubation with the Brightvision goat anti-rabbit HRP (Cat# DPVR-HRP, Immunologic) was conducted. After washing, the sections were stained for 10 min with BrightDAB (catalog no. BS04, Immunologic). Finally, tissue sections were counterstained with Hematoxylin and cover-slipped.

### Single-cell mRNA sequencing

PDOs were cultured in Matrigel for 6 days and subsequently seeded into a Collagen network (invasive) or back into Matrigel (non-invasive) for a further 3 days. The invasive PDOs were subjected to Collagenase (catalog no. C0130, Sigma-Aldrich) treatment for 3 min. at 37 °C. Next, the invasive and non-invasive PDOs were trypsinized for 10 min. to dissociate organoids into single cells. Single cells were resuspended in FACS buffer (0.5 mM EDTA and 5% FCS) and individual viable cell (DAPI negative) were seeded into three 384 well plates per condition. Single-cell mRNA-sequencing was performed by Single Cell Discoveries (Utrecht, The Netherlands) and downstream analysis was processed, explored and visualized using Cellenics® community instance (https://scp.biomage.net/) hosted by Biomage (https://biomage.net/). Briefly, cells with low quality reads, as determined by cells exhibiting ≤1000 unique molecular identifiers (UMIs) and mitochondrial reads higher than 5%, were filtered out. MASS package (v. 7.3–56) was applied to determine the linear relationship between genes with at least one count, and the UMIs of each barcode. Next, the predicted gene number for each barcode was determined using a fitted model tolerance of 1–α, where α is 1 divided by the number of droplets in each sample, and the predicted cells inside the upper and lower boundaries were retained. scDblFinder (R package v. 1.11.37) was used to determine the probability of droplets containing duplicate cells. A duplicate score of >0.5 was applied to remove these cells from further analysis. To integrate data, ‘Harmony’ was applied. Clustering was performed using Seurat’s implementation of the Louvain method. Adjusted *p* value was determined using the using the Benjamini–Hochberg correction. To generate focused invasion signatures, cell cycle-related genes (KEGG_CELL_CYCLE) were excluded from further analysis.

### Gene ontology and GSEA

Differentially expressed genes (logFC ≥ 0.5*,* Adj*. p* < 0.001) derived from the single cell mRNA sequencing were subjected to GSEA using Enrichr [57–59]. Publicly available gene set pathways used to generate both invasion signatures were: cell motility GO:0048870, cell adhesion GO:0007155, cell adhesion mediated by integrin GO:0033627, positive regulation of cell–cell adhesion GO:0022409, positive regulation of cell-substrate adhesion GO:0010811, cell-matrix adhesion GO:0007160, extracellular matrix organization GO:0030198, amoeboid-type cell migration GO:0001667, positive regulation of cell migration GO:0030335.

### Clinical cohort analysis

The Head and Neck Cancer TCGA dataset underwent filtering to exclude HPV positive patients, resulting in a cohort of 438 patients. Next, hierarchical clustering was used to identify genes with strong cross-correlation patterns, enabling us to minimize confounding effects and enhance the signature’s specificity. The H&N cancer validation cohort (GSE65858 [32]) consisted of 196 patients once HPV positive patients were filtered out. Firstly, the gene expression matrix was filtered on the bases of probe detection *p* value, and genes with a high number of missing values were removed (>70%). Subsequently, the missing values were replaced with random numbers coming from the 15th percentile, and data were normalized using normalize.quantiles function of preprocessCore package (version 1.66.0). Upon z-score calculation per gene, we removed genes with low variability (Standard Deviation <0.5) and calculated activity scores per patient using the 7 remaining genes (*THBS1*, *ADAMTS1*, *CSF2*, *PDPN*, *SERPINE1*, *SPARC*, and *TNC*). Similarly, the full collective signature was further investigated in all major solid cancer groups (29 excluding H&N cancer) in the TCGA dataset. For each of the cohorts described above, survival analysis was performed using Overall Survival (OS) parameter comparing the group with high activity score and the one with low activity score (mean value of z-scores of all genes per patient; split in two halves containing the same number of cases). Hazard ratios (HRs) and 95% confidence intervals for survival curves were calculated using a Cox proportional hazards model. All calculations were done in R.

### YAP quantification

Confocal images of Collagen embedded PDOs were immunofluorescence labeled for YAP and assessed for nuclear and cytosolic expression using CellProfiler [60]. An individual confocal slice from the center of an organoid was selected and subjected to cell segmentation and fluorescence intensity quantification. Twelve individual organoid structures were quantified over 3 separate experiments. Cells that were incorrectly segmented, were removed from further analysis. Next, YAP activity was determined by dividing nuclear over cytosolic YAP intensity. Cells were manually assigned positional information: collective strand (all cells touching the cell-ECM interface in a strand), invasive single cells (cells that have dissociated from the PDO) or core (inner layer of cells/not in contact with ECM). Similarly, nuclear/cytosolic ratios for IHC stained primary tissue sections was determined using QuPath (version 0.4.4). Invasive strands, single cells or tumor core cells (inner layer of cells, excluding the outer 3 layers of cells proximal to the ECM) were segmented based of optical density followed by quantification of the DAB signal. For whole tissue sections corresponding to the collective and single cell invasive PDO models, 5 individual regions (250 µm × 250 µm) at the tumor invasive front or tumor core were quantified per sample. Validation of these findings were performed on 10 collective and 10 single cell invasive YAP IHC stained sections, whereby 3 separate regions were quantified at the tumor invasive front. GraphPad Prism was used for statistical analysis. Statistical significance was calculated using the unpaired Mann–Whitney or Kruskal–Wallis test.

### Collagen-I alignment analysis

OrientationJ, a publicly available ImageJ plugin determined the local orientation (range from −90° to +90°) and coherency (ranges from 0 to 1) of every pixel in a selected region of interest. The local orientation and coherency values were analyzed using the custom-made ImageJ plugin, Quantify Remodeling (developed by ref. [17]), so that Collagen fibers perpendicular to the invasive cell-ECM border could be analyzed. Collagen fibers perpendicular to 10 invasive structures were quantified in triplicate per condition. Perfect fiber alignment is defined as 1, and the cut-off for non (random)-alignment is 0.2.

### Supplementary information

Supplementary_Material

Supplementary_Table_1

Supplementary_Table_2

Supplementary_Table_3

Supplementary_Table_4

## Data Availability

Single cell mRNA sequencing data reported in this paper have been deposited in the NCBI Gene Expression Omnibus (GEO) database, www.ncbi.nlm.nih.gov/geo (accession no. GSE244956).
