# Supplementary material for: Differential transcriptional invasion signatures from patient derived organoid models define a functional prognostic tool for head and neck cancer
Source: Oncogene. 2024 Jun 28;43(32):2463–74. doi: 10.1038/s41388-024-03091-4 (PMC11315671; doi:10.1038/s41388-024-03091-4)
Supplement: Supplementary file 1 — Supplementary_Material [file 41388_2024_3091_MOESM1_ESM.pdf]

Supplemental Table Legends

Supplementary Table 1: Differential expression analysis from single cell mRNA sequencing of invasive (Collagen) versus non-invasive (Matrigel) models

Supplementary Table 2: Differential expression analysis from single cell mRNA sequencing of collective versus single cell invasive models

Supplementary Table 3: GSEA of single cell invasion according to GO term: Biological Processes

Supplementary Table 4: GSEA of collective invasion according to GO term: Biological Processes

Supplemental Figure Legends

Supplemental Figure 1. Invasive HNSCC PDO models and the spatial expression pattern of basal marker, Keratin 14.

A) Collagen-embedded HNSCC PDO models (left panels) and their corresponding primary tumor sections (right panels) were analyzed for K14 protein (Basal marker; green) expression using immunofluorescence. Merged images show K14 (green), Collagen-I (white) and DNA (blue, DAPI). Arrow heads indicate invasive structures. Scale bars: 200 µm. B) Additional HNSCC PDO models T2, T6, T257 and T276, cultured in Matrigel (non-invasive, top panels) or Collagen (invasive, bottom panels) showing mixed invasive phenotypes. Black arrow heads indicate mixed invasive modes. Scale bars: 20 µm.

Supplemental Figure 2. Single cell mRNA sequencing analysis of invasive and non-invasive conditions in the HNSCC PDO models

A-C) Single cell mRNA sequencing scatter plots showing the read counts (A), UMI counts (B) and mitochondrial fractions (C) for individual cells after cut-offs were applied. D) Heatmap showing scaled expression of epithelial genes in non-invasive and invasive conditions from single cell transcriptomics. E) t-SNE plot of color-coded clusters defined using the Louvain clustering method. F-H) Expression profiles of established invasion related genes identified in HNSCC. Shown are t-SNE plots (upper panels) and violin plots for normalized mRNA expression (lower panels) of *ITGA2*, *DDR1* and *KRT14*.

Supplemental Figure 3. ECM adhesion regulation and transcriptomic clustering of single cell and collective invasive HNSCC PDO models

A) β-catenin expression in the primary tumors corresponding to the collective or single cell invasive HNSCC PDO models. Arrows indicate invasive strands or single cells in the respective models. Scale bars: 200 µm. B) tSNE plot showing Louvain clustering of single cell mRNA sequencing data from collective and single cell invasive models embedded in Collagen. C-D) Heatmaps displaying combined single cell transcriptomic expression of integrins (C), and top 20 differentially expressed genes associated with the ‘extracellular matrix organization’ (D) or ‘extracellular matrix structural constituent’ (E) GO terms for the collective and single cell invasion models.

Supplemental Figure 4. YAP activation correlates with Collagen alignment during collective invasion

A-B) Collective and single cell models seeded in Collagen for 72 hrs. were stained for DNA (DAPI, blue) and the collagen matrix was immunofluorescence labeled using a CNA probe (gray) (upper panel, A). Scale bars: 50 µm. Insets indicate pseudo coloring of Collagen-I fiber orientation angles from −90° to 90°. B) Collagen fiber alignment from (A) was quantified in (B) for the collective and single cell models. Collagen fibers perpendicular to 10 invasive structures was quantified in triplicate per a condition. Y-axis represents the degree of collagen alignment and x-axis the alignment length. Perfect fiber alignment is defined as 1, and the cut-off for non (random)-alignment is 0.2. Error bars indicate SD; ns indicates non-significant; $*p < 0.05$, $**p < 0.01$. Statistical significance was calculated using the Kruskal–Wallis test.

Supplemental Figure 5. Collective signature provides prognostic utility in multiple cancer types

A) Prognostic utility of the collective invasive signature in all solid cancer groups. High and low expression groups from the TCGA database were defined by z-scores and compared using a log-rank test. The graph represents an overview of patient median survival time in each cancer group, where the x-axis represents time (days) to 50% survival, and the y-axis indicate the cancer type (left) and statistical significance (right). Groups that did not reach 50% survival probability according to the collective invasive signature are highlighted in blue. Cancers for which neither of the groups reached 50% survival are not depicted. B-H) Cancer types that demonstrate prognostic statistical significance using the collective invasive signature that reached 50% survival probability from panel B (C-H), or was significant but did not reach 50% survival probability threshold (I) are presented as Kaplan-Meier survival curves. All Kaplan-Meier survival curves depict the probability of survival over time, where the x-axis represents time in days, and the y-axis indicates the proportion of survival. Statistical significance was determined by p-value calculation. Hazard ratios (HRs) and 95 % confidence intervals for survival curves were calculated using a Cox proportional hazards model.

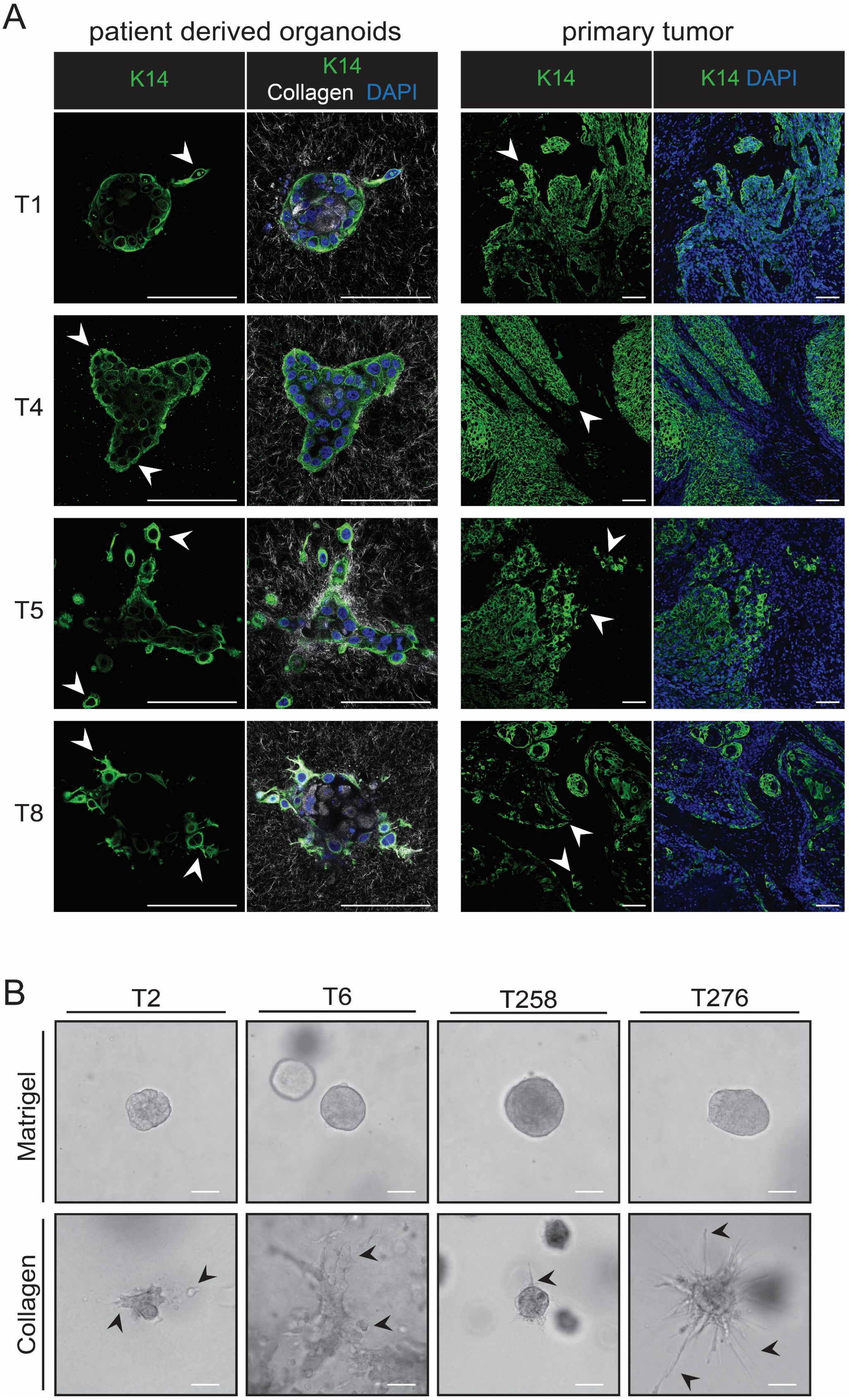
A
patient derived organoids
primary tumor
K14
K14
Collagen DAPI
K14
K14 DAPI
T1
T4
T5
T8
B
T2
T6
T258
T276
Matrigel
Collagen

Sup 2

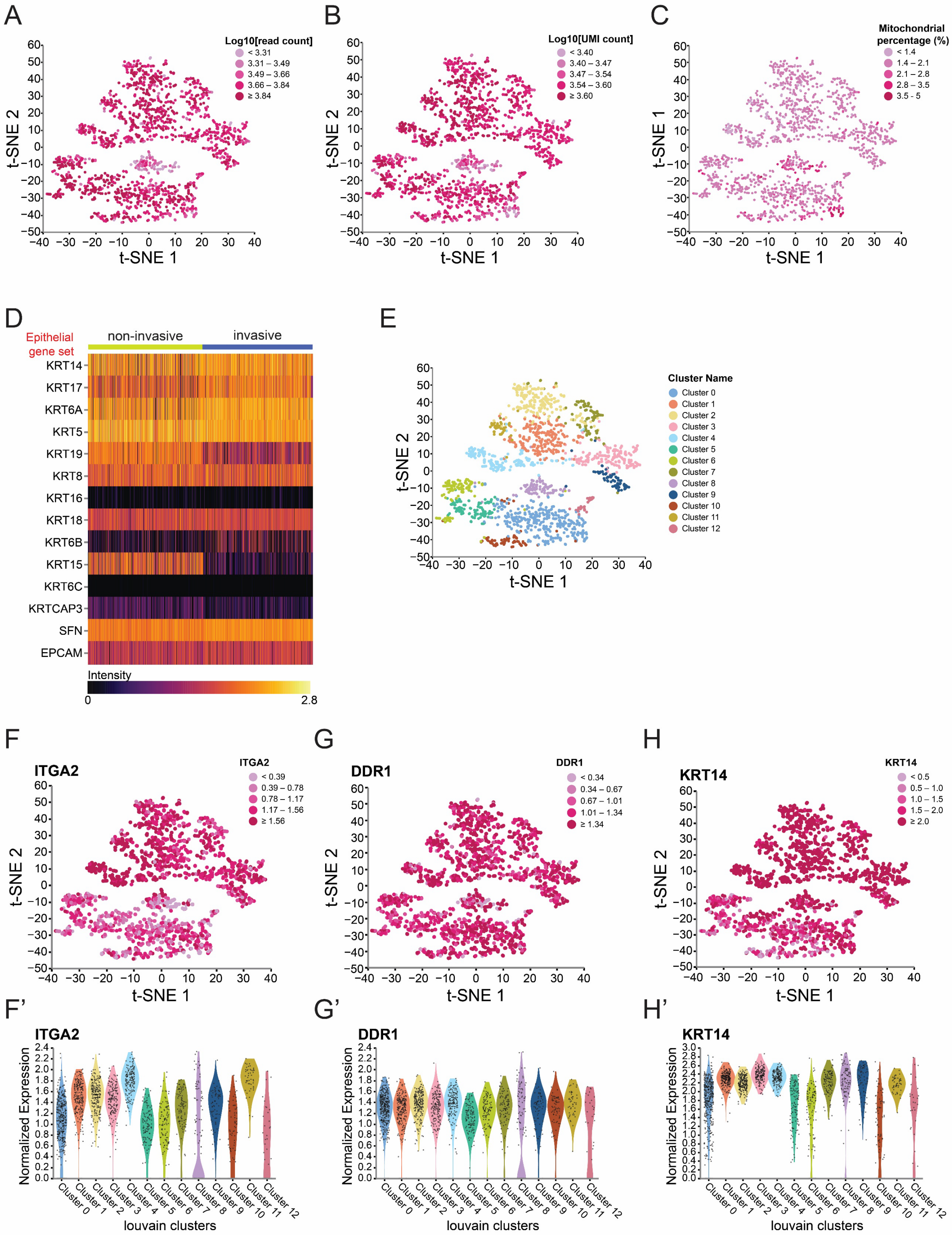
A
Log10[read count]
< 3.31
3.31 – 3.49
3.49 – 3.66
3.66 – 3.84
≥ 3.84
t-SNE 2
t-SNE 1
B
Log10[UMI count]
< 3.40
3.40 – 3.47
3.47 – 3.54
3.54 – 3.60
≥ 3.60
C
Mitochondrial percentage (%)
< 1.4
1.4 – 2.1
2.1 – 2.8
2.8 – 3.5
3.5 - 5
t-SNE 1
D
Epithelial gene set
non-invasive
invasive
KRT14
KRT17
KRT6A
KRT5
KRT19
KRT8
KRT16
KRT18
KRT6B
KRT15
KRT6C
KRTCAP3
SFN
EPCAM
Intensity
0
2.8
E
Cluster Name
Cluster 0
Cluster 1
Cluster 2
Cluster 3
Cluster 4
Cluster 5
Cluster 6
Cluster 7
Cluster 8
Cluster 9
Cluster 10
Cluster 11
Cluster 12
F
ITGA2
< 0.39
0.39 – 0.78
0.78 – 1.17
1.17 – 1.56
≥ 1.56
G
DDR1
< 0.34
0.34 – 0.67
0.67 – 1.01
1.01 – 1.34
≥ 1.34
H
KRT14
< 0.5
0.5 – 1.0
1.0 – 1.5
1.5 – 2.0
≥ 2.0
F'
G'
H'
Normalized Expression
louvain clusters

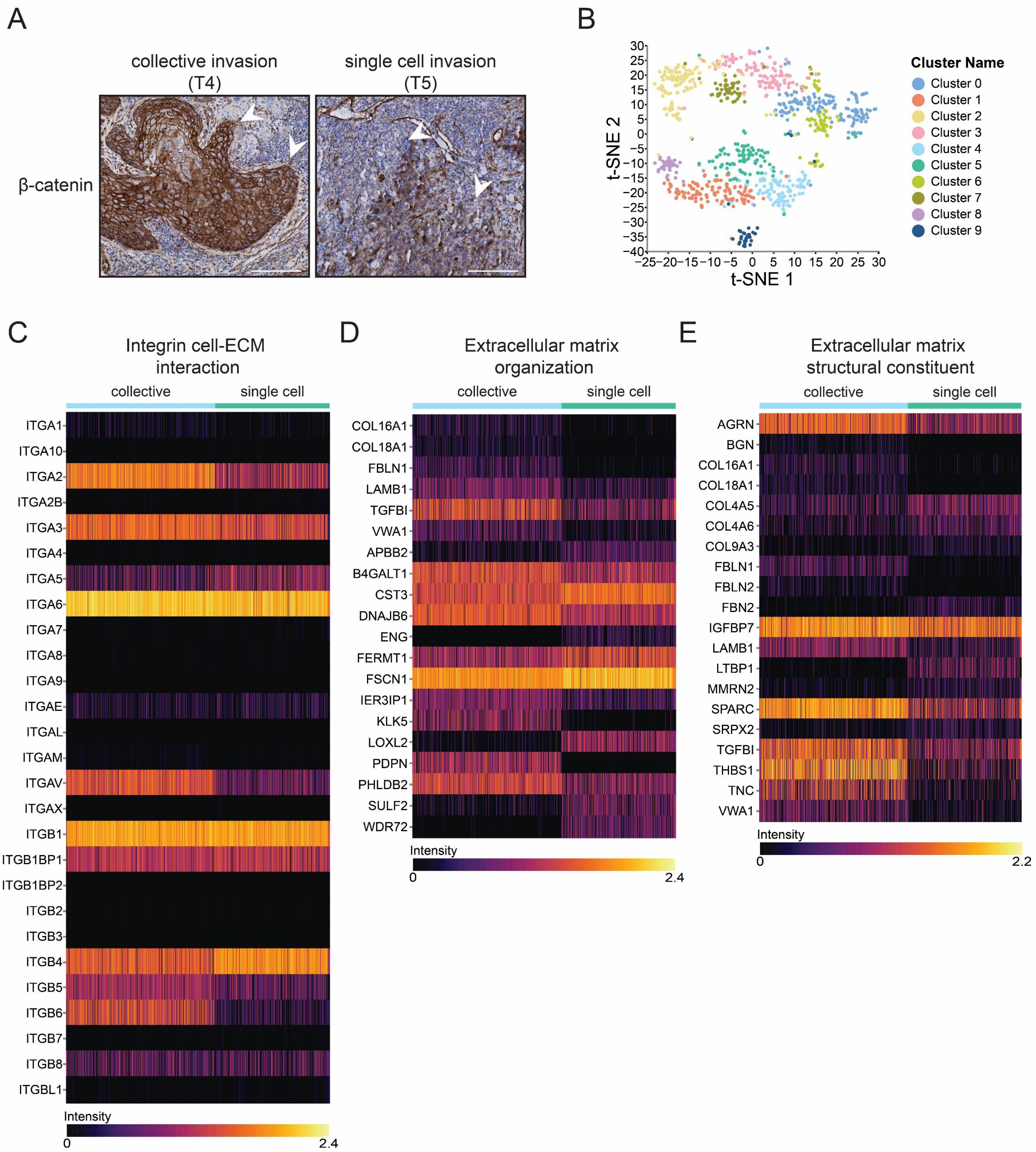
A
collective invasion (T4)
single cell invasion (T5)
β-catenin
B
t-SNE 2
t-SNE 1
30 25 20 15 10 5 0 −5 −10 −15 −20 −25 −30 −35 −40
−25 −20 −15 −10 −5 0 5 10 15 20 25 30
Cluster Name
Cluster 0
Cluster 1
Cluster 2
Cluster 3
Cluster 4
Cluster 5
Cluster 6
Cluster 7
Cluster 8
Cluster 9
C
Integrin cell-ECM interaction
collective
single cell
ITGA1
ITGA10
ITGA2
ITGA2B
ITGA3
ITGA4
ITGA5
ITGA6
ITGA7
ITGA8
ITGA9
ITGAE
ITGAL
ITGAM
ITGAV
ITGAX
ITGB1
ITGB1BP1
ITGB1BP2
ITGB2
ITGB3
ITGB4
ITGB5
ITGB6
ITGB7
ITGB8
ITGBL1
Intensity
0
2.4
D
Extracellular matrix organization
collective
single cell
COL16A1
COL18A1
FBLN1
LAMB1
TGFBI
VWA1
APBB2
B4GALT1
CST3
DNAJB6
ENG
FERMT1
FSCN1
IER3IP1
KLK5
LOXL2
PDPN
PHLDB2
SULF2
WDR72
Intensity
0
2.4
E
Extracellular matrix structural constituent
collective
single cell
AGRN
BGN
COL16A1
COL18A1
COL4A5
COL4A6
COL9A3
FBLN1
FBLN2
FBN2
IGFBP7
LAMB1
LTBP1
MMRN2
SPARC
SRPX2
TGFBI
THBS1
TNC
VWA1
Intensity
0
2.2

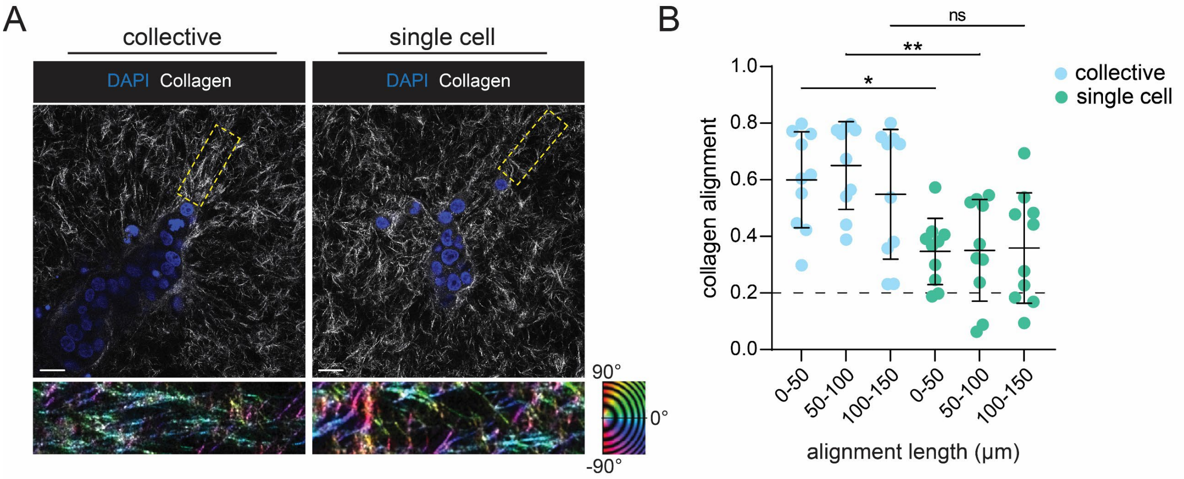
A
collective
single cell
DAPI Collagen
DAPI Collagen
90°
0°
-90°
B
ns
**
*
1.0
0.8
0.6
0.4
0.2
0.0
collagen alignment
0-50
50-100
100-150
0-50
50-100
100-150
alignment length (μm)
collective
single cell

Sup 5

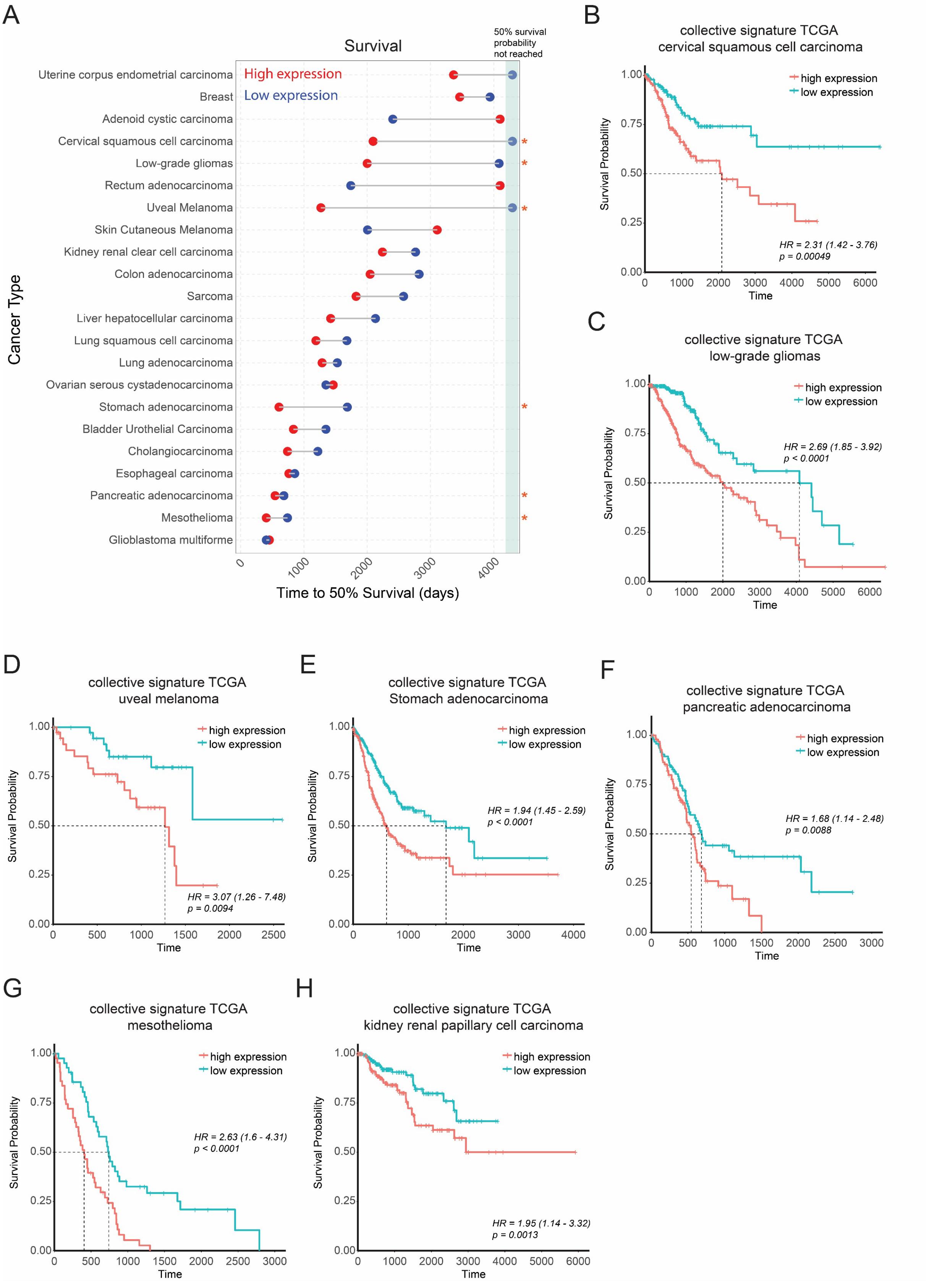
A
Survival
50% survival probability not reached
High expression
Low expression
Cancer Type
Uterine corpus endometrial carcinoma
Breast
Adenoid cystic carcinoma
Cervical squamous cell carcinoma
Low-grade gliomas
Rectum adenocarcinoma
Uveal Melanoma
Skin Cutaneous Melanoma
Kidney renal clear cell carcinoma
Colon adenocarcinoma
Sarcoma
Liver hepatocellular carcinoma
Lung squamous cell carcinoma
Lung adenocarcinoma
Ovarian serous cystadenocarcinoma
Stomach adenocarcinoma
Bladder Urothelial Carcinoma
Cholangiocarcinoma
Esophageal carcinoma
Pancreatic adenocarcinoma
Mesothelioma
Glioblastoma multiforme
Time to 50% Survival (days)
B
collective signature TCGA cervical squamous cell carcinoma
high expression
low expression
HR = 2.31 (1.42 - 3.76)
p = 0.00049
Survival Probability
Time
C
collective signature TCGA low-grade gliomas
HR = 2.69 (1.85 - 3.92)
p < 0.0001
D
collective signature TCGA uveal melanoma
HR = 3.07 (1.26 - 7.48)
p = 0.0094
E
collective signature TCGA Stomach adenocarcinoma
HR = 1.94 (1.45 - 2.59)
p < 0.0001
F
collective signature TCGA pancreatic adenocarcinoma
HR = 1.68 (1.14 - 2.48)
p = 0.0088
G
collective signature TCGA mesothelioma
HR = 2.63 (1.6 - 4.31)
p < 0.0001
H
collective signature TCGA kidney renal papillary cell carcinoma
HR = 1.95 (1.14 - 3.32)
p = 0.0013
